# Documented Dry Eye Disease in Patients Receiving Topical Antiglaucoma Therapy: Associations with Treatment Characteristics

**DOI:** 10.3390/biomedicines14102344

**Published:** 2026-10-15

**Authors:** Marija Jelić Vuković, Ena Kolak, Dubravka Biuk, Petar Šušnjara, Stipe Vidović, Eleonora Suvaljko, Leon Perić, Vedran Nemet, Luka Prce, Suzana Matić

**Affiliations:** 1 Department of Ophthalmology, University Hospital Center Osijek, 31 000 Osijek, Croatia; marija.jelic.vukovic@gmail.com (M.J.V.); dubravka.biuk@gmail.com (D.B.); stipevidovic1@gmail.com (S.V.); lperic1@yahoo.com (L.P.); nemet.vedran@gmail.com (V.N.); suzimatic72@gmail.com (S.M.); 2 Faculty of Medicine, Josip Juraj Strossmayer University of Osijek, 31 000 Osijek, Croatia; 3 Faculty of Kinesiology Osijek, Josip Juraj Strossmayer University of Osijek, 31 000 Osijek, Croatia; psusnjara@kifos.hr; 4 Department of Ophthalmology, General Hospital Virovitica, 33 000 Virovitica, Croatia; nora.suvaljko@gmail.com; 5 School of Medicine, University of Zagreb, 10 000 Zagreb, Croatia; luka.prce@gmail.com

**Keywords:** glaucoma, dry eye disease, ocular surface disease, topical antiglaucoma therapy, medication burden, polypharmacy, preservative-free regimen, benzalkonium chloride

## Abstract

**Background/Objectives:** Chronic topical antiglaucoma therapy may affect the ocular surface. We assessed documented dry eye disease (DED) and its associations with active-ingredient burden, treatment duration, and preservative exposure. **Methods:** This retrospective cross-sectional study included 357 adults receiving topical antiglaucoma therapy at a tertiary center who had complete treatment and ocular-surface data. Ocular-surface testing was prompted by symptoms or clinical judgment. The first eligible visit identified during chronological record review was analyzed for each patient. DED was based on the treating ophthalmologist’s documented diagnosis. Logistic regression evaluated treatment-related factors. **Results:** DED was documented in 171/357 patients within this selected sample (47.9%; 95% CI 42.8–53.1%). Prevalence was higher with at least two versus one active IOP-lowering ingredient (60.6% vs. 23.1%; *p* < 0.001). Each additional ingredient was associated with higher DED odds after adjustment for age, sex, and glaucoma subtype (adjusted odds ratio [aOR] 2.65, 95% confidence interval [CI] 1.88–3.74). Longer treatment duration was also associated with higher odds (aOR 1.12 per 12 months, 95% CI 1.05–1.20). In the linear extended model, active-ingredient count was not associated with documented DED (aOR 1.02, 95% CI 0.69–1.51), whereas an exclusively preservative-free regimen was associated with lower odds (aOR 0.011, 95% CI 0.001–0.084) and fixed-combination therapy with higher odds (aOR 3.23, 95% CI 1.74–5.98). However, categorical modeling of ingredient count significantly improved extended-model fit (likelihood-ratio *p* < 0.001), indicating sensitivity to functional form. Documented DED occurred in 1/91 (1.1%) exclusively preservative-free patients versus 170/266 (63.9%) preserved-exposure patients, with sparse PF events limiting interpretation. **Conclusions:** Documented DED was common in this selected cohort undergoing ocular-surface assessment. Active-ingredient count was associated with DED in the primary adjusted model, but its interpretation in the extended model depended on the functional form used. The marked inverse association with an exclusively preservative-free regimen requires prospective confirmation because sparse data, limited overlap between treatment groups, treatment selection, and residual confounding preclude causal inference.

## 1. Introduction

Glaucoma is the leading cause of irreversible blindness worldwide and is a chronic condition that frequently requires lifelong treatment to reduce intraocular pressure and prevent progressive optic nerve damage and visual field loss [1]. Topical intraocular pressure-lowering medications remain a central component of glaucoma management and are often administered for prolonged periods, either as monotherapy or in multidrug regimens. However, chronic topical treatment may adversely affect the ocular surface and contribute to ocular discomfort, tear film instability, and dry eye disease (DED) [2].

Dry eye disease is a multifactorial disease of the ocular surface characterized by loss of tear film homeostasis and accompanied by ocular symptoms, in which tear film instability, hyperosmolarity, ocular surface inflammation and damage, and neurosensory abnormalities play etiological roles [3]. According to the TFOS DEWS II Epidemiology Report, the prevalence of DED ranges from approximately 5% to 50%, depending on the population and diagnostic criteria applied [4]. In addition to ocular discomfort, DED may impair visual function and quality of life. Among patients receiving topical glaucoma therapy, ocular surface abnormalities have been associated with greater daily instillation burden and poorer symptom- and vision-related quality of life [5].

Several clinical studies have demonstrated a high prevalence of DED and abnormal ocular surface parameters among patients receiving topical antiglaucoma therapy. Increasing age, longer treatment duration, greater medication burden, and exposure to preserved formulations have been associated with worse OSDI scores, reduced tear film breakup time (TBUT), lower Schirmer test values, and other signs of ocular surface dysfunction [6,7,8,9]. However, reported prevalence estimates vary considerably. Kobia-Acquah et al. reported a DED prevalence of 23% in treated glaucoma patients, whereas Ramli et al. found ocular surface disease prevalence estimates ranging from 37% to 91%, depending on the diagnostic test applied [6,7]. In a large retrospective study of 1274 patients, Küçük et al. identified documented dry eye and artificial tear use in 27% of patients receiving topical antiglaucoma medications [9]. These differences illustrate the substantial influence of study population, outcome definition, and diagnostic methodology on reported DED prevalence.

Ocular surface toxicity related to topical glaucoma treatment may result from preservatives, active pharmacological compounds, or their combined effects [2]. Benzalkonium chloride (BAK), one of the most frequently used preservatives in ophthalmic formulations, has been associated with oxidative stress, epithelial and conjunctival goblet cell toxicity, altered mucin expression, inflammation, and reduced tear film stability [10,11]. Treatment intensity may further increase cumulative exposure to preservatives and active compounds. Clinical studies have reported associations between a greater number of active IOP-lowering ingredients or daily instillations and worse ocular surface symptoms and signs [6,7,8]. Conversely, preservative-free formulations have been proposed as a means of reducing treatment-related ocular surface toxicity, although the available evidence remains heterogeneous and is influenced by differences in treatment regimens, diagnostic criteria, and study design [12].

Despite increasing recognition of treatment-related ocular surface disease, important uncertainties remain regarding the relative contributions of medication burden, treatment duration, and formulation type in routine clinical practice. Many available studies are cross-sectional, use different definitions of DED, or evaluate selected ocular surface outcomes rather than combining documented clinical diagnoses with treatment characteristics and complementary symptom- and sign-based measures. Additional real-world evidence from diverse clinical populations may therefore improve understanding of the factors associated with ocular surface disease among patients receiving long-term topical glaucoma treatment.

The present study aimed to determine the prevalence of documented DED and examine its associations with medication burden, treatment duration, and preservative exposure in a retrospective cross-sectional sample of patients receiving topical antiglaucoma treatment at a tertiary ophthalmology center. We hypothesized that greater medication burden and longer treatment duration would be associated with a higher prevalence of documented DED and less favorable ocular surface parameters, whereas exclusively preservative-free regimens would be associated with a lower prevalence of documented DED.

## 2. Materials and Methods

### 2.1. Study Design and Setting

This retrospective cross-sectional observational study was conducted at the Department of Ophthalmology, University Hospital Centre Osijek, Croatia. Ambulatory ophthalmology reports in the institutional electronic medical record system were manually reviewed in chronological order, from earlier to later encounters, for the period from 1 January 2021 to 1 January 2026. Patients with glaucoma were identified through review of the clinical reports, and eligible data were manually entered into the analytical dataset. For each patient, the first encounter meeting the eligibility criteria and containing complete contemporaneous treatment and ocular-surface data was selected as the index visit. Subsequent encounters for patients already included were skipped. Each patient therefore contributed one observation and was the unit of analysis. The study was reported in accordance with the Strengthening the Reporting of Observational Studies in Epidemiology (STROBE) statement [13].

### 2.2. Participants

The study included adults with confirmed glaucoma who had received continuous topical antiglaucoma therapy for at least three months. Inclusion required complete contemporaneous treatment information, documented DED status, TBUT, Schirmer test values, and OSDI scores at the index visit. Ocular-surface assessments were performed according to symptoms or the treating ophthalmologist’s clinical judgment, rather than systematically in all glaucoma patients. Consequently, the analytical sample was restricted to patients who underwent these assessments and had complete records. Patients were excluded if they had previous corneal or ocular-surface surgery likely to independently affect ocular-surface integrity, an active ocular-surface infection at the analyzed assessment, or incomplete or unreliable medical records. Previous oculoplastic surgery was recorded separately and was not an exclusion criterion.

No a priori sample-size calculation was performed; all eligible patients meeting the predefined criteria during the study period were included.

### 2.3. Data Collection

Extracted variables comprised age, sex, glaucoma subtype, duration of documented topical treatment, number of active intraocular pressure (IOP)-lowering ingredients used at the index visit, treatment class (prostaglandin analogue, beta-blocker, alpha-agonist, carbonic anhydrase inhibitor, and fixed-combination therapy), exclusively PF regimen status, artificial-tear use, diabetes mellitus, hypertension, and previous oculoplastic surgery. Each active component of a fixed-dose combination was counted separately when determining active-ingredient burden. Ocular-surface variables included documented DED status, TBUT, Schirmer test values, and OSDI scores. Patients were classified as receiving an exclusively PF regimen only when all topical antiglaucoma formulations used at the index visit were preservative-free; regimens containing at least one preserved formulation were classified as preserved-exposure regimens. Artificial-tear formulations were not used to classify antiglaucoma preservative exposure. The analytical dataset did not distinguish whether the recorded index-visit regimen represented treatment used before the examination or prescriptions issued afterward. Consequently, same-visit treatment changes in response to ocular-surface findings could not be excluded. Information on preservative type and concentration, the number of preserved formulations per regimen, and dosing frequency was not available in the analytical dataset. Consequently, the classification reflects preservative exposure status at the index visit rather than quantitative or cumulative preservative burden. Preservative exposure was classified according to the topical antiglaucoma regimen documented at the index visit. Information on prior exposure to preserved formulations, treatment switching, and the clinical reasons for prescribing an exclusively preservative-free regimen was not available in the analytical dataset. Therefore, current regimen status should not be interpreted as cumulative preservative exposure.

Drug-class counts were tabulated from the recorded binary class indicators and were not mutually exclusive. Fixed-combination use was tabulated separately and overlapped with the recorded drug classes. In the analytical dataset, all fixed-combination users also had prostaglandin-analogue and beta-blocker indicators; these patients were therefore included in the corresponding class counts in Table 1 and Table 2. Named prescriptions were not available in the analytical dataset for independent verification of individual components.

Treatment duration referred to the total documented duration of topical antiglaucoma therapy available in the clinical record, rather than the duration of the specific regimen recorded at the index visit. Changes in active ingredients, formulations, or treatment regimens during this period could not be reconstructed from the analytical dataset. Complete information on treatment received before presentation to University Hospital Centre Osijek could not be verified for all patients; therefore, this variable represents documented treatment duration rather than necessarily lifetime cumulative exposure.

Demographic, treatment-related, and ocular-surface data were obtained from the same index-visit record. TBUT and Schirmer test results were retained as patient-level entries without ocular laterality and were analyzed as recorded. The analytical dataset did not specify whether these entries represented one eye, the worse eye, or a summary of both eyes. No bilateral averaging or eye selection was performed during analysis. Consequently, eye-specific measurements could not be reconstructed, no separate eye-level analyses were performed, and potential inter-eye differences could not be examined.

Ocular-surface measurements were extracted exactly as documented, without retrospective transformation, interpolation, or imputation. TBUT and Schirmer test results were recorded as integer values, and OSDI scores were retained as recorded scores. Item-level OSDI responses were not available for independent recalculation. The origin of the frequently repeated values could not be established from the analytical dataset. Data were pseudonymized before analysis, and only aggregated results are reported.

### 2.4. Assessment of Ocular Surface Disease

Dry eye disease (DED) status was determined from the diagnosis recorded by the treating ophthalmologist in the institutional electronic medical record at the index visit. Patients were classified as DED when a clinical diagnosis of dry eye disease was documented at the index visit and as non-DED when no such diagnosis was recorded. The term “non-DED” therefore denotes absence of a documented diagnosis rather than an explicit negative clinical assessment. Artificial-tear use and individual OSDI, TBUT, or Schirmer values were not used to retrospectively override this classification. The available records did not allow us to verify whether all ophthalmologists used common diagnostic criteria, uniform test cut-offs, or the same sequence of ocular-surface assessments during the study period. Therefore, the documented clinical diagnosis was used as the primary outcome. Variation in routine diagnostic practice may have resulted in misclassification of DED status.

Tear film breakup time (TBUT), Schirmer test values, and Ocular Surface Disease Index (OSDI) scores were available for all included patients and were analyzed as complementary ocular-surface measures. These assessments were performed according to symptoms or the treating ophthalmologist’s clinical judgment, rather than systematically in all glaucoma patients. Their availability for all included patients reflects the requirement for complete ocular-surface data for inclusion. This requirement may have preferentially selected patients undergoing more extensive ocular-surface assessment. Corneal or conjunctival staining scores, tear film osmolarity, and meibomian gland assessment were not available in the analytical dataset. TBUT values were additionally categorized as <5 s, 5–10 s, and >10 s for descriptive analyses of tear film stability. These categories were not used to establish the primary DED diagnosis.

The analytical dataset did not retain details of the TBUT technique, including fluorescein use or repeat-measurement averaging, Schirmer test duration and anesthesia use, or the OSDI language/version and original scoring procedure. These aspects of measurement were not independently verified against source records, and a uniform measurement protocol cannot be assumed. Recorded patient-level TBUT and Schirmer values were used without retrospective selection of an eye or construction of a bilateral summary.

### 2.5. Statistical Analysis

Continuous variables are presented as medians and interquartile ranges (IQRs), and categorical variables as counts and percentages. Distributional assumptions were assessed using histograms, Q–Q plots, skewness and kurtosis, and the Shapiro–Wilk test. Because continuous variables were non-normally distributed and several ocular-surface measures showed clustering at commonly recorded values, non-parametric methods were used. Two-group comparisons employed the Mann–Whitney U test for continuous variables and the chi-square or Fisher exact test for categorical variables, as appropriate. Correlations were evaluated using Spearman rank coefficients.

Wilson score 95% confidence intervals were calculated for the principal proportions of documented DED. Percentages describing treatment use within DED outcome groups were distinguished from DED proportions within treatment groups. Treatment duration was modeled continuously; its odds ratio is additionally expressed per 12 months by exponentiating the fitted coefficient and its confidence limits multiplied by 12. The previous 12-month dichotomy was removed from the main descriptive comparison because the original basis for that cutoff was not documented and the resulting groups were markedly unequal. All six combinations of the two treatment variables and three ocular-surface measures were reported together.

Multivariable binary logistic regression evaluated factors associated with documented DED. Because treatment duration and active-ingredient count were strongly correlated, they were not included simultaneously in the primary models. Model 1 included age, sex, glaucoma subtype, and active-ingredient count. Model 2 included age, sex, glaucoma subtype, and treatment duration. An extended treatment-related model included age, sex, glaucoma subtype, active-ingredient count, exclusively PF regimen status, fixed-combination therapy, hypertension, and diabetes mellitus; treatment duration was omitted because of its strong correlation with active-ingredient count. Results are reported as adjusted odds ratios (aORs) with 95% confidence intervals (CIs). Primary open-angle glaucoma, male sex, preserved-exposure regimen, absence of fixed-combination therapy, absence of hypertension, and absence of diabetes were the reference categories.

Sensitivity analyses assessed the functional form of active-ingredient count and documented treatment duration. Active-ingredient count was entered as a categorical variable, with one ingredient as the reference, in Model 1 and the conventional extended model. These models were compared with their linear-count counterparts using likelihood-ratio tests with two degrees of freedom. In Model 2, treatment duration was modeled using a restricted cubic spline with three knots at the 10th, 50th, and 90th percentiles (14.6, 65.0, and 154.4 months). Departure from linearity was assessed using a likelihood-ratio test with one degree of freedom. All comparisons retained the same adjustment covariates and analytical sample. These exploratory analyses used conventional maximum-likelihood estimation in Python version 3.12.14 with SciPy version 1.17.0.

An exploratory supplementary analysis was restricted to patients receiving at least two active IOP-lowering ingredients. Conventional maximum-likelihood logistic regression evaluated fixed-combination use, adjusting for categorical ingredient count, age, sex, glaucoma subtype, hypertension, and diabetes mellitus. Exclusively PF regimen status was omitted because all patients in this subset received preserved-exposure regimens. Treatment duration was omitted, consistent with the original extended-model specification. Two-sided Wald *p* values and 95% confidence intervals were calculated using the inverse observed-information matrix. This supplementary analysis was performed in Python using SciPy.

Because only one DED event occurred among patients receiving an exclusively PF regimen, Firth bias-reduced penalized logistic regression using the same covariates as the conventional linear extended model was performed as a sensitivity analysis. For this revision, the analysis was rerun using a documented Python implementation that maximized the ordinary log likelihood plus one-half of the log determinant of the expected information matrix. Standard errors were obtained from the inverse ordinary expected-information matrix evaluated at the Firth estimates. Confidence intervals were calculated as exp(β ± 1.96 SE), and two-sided *p* values used Wald z tests; profile-penalized likelihood intervals and tests were not used. Numerical checks assessed the adjusted-score residual (maximum absolute value <10^−8^), positive definiteness of the information and numerical objective-Hessian matrices, agreement between two starting values within 10^−5^ per coefficient, and agreement of the analytic gradient with finite differences. BFGS fitting was followed by score-equation refinement where numerical precision warnings occurred. The core code and diagnostic output are provided as Supplementary Code 1. An additional analysis excluded patients with previous oculoplastic surgery and refitted both the conventional and Firth models with unchanged covariates.

Descriptive analyses and the original conventional regression analyses were performed using IBM SPSS Statistics version 27.0 (IBM Corp., Armonk, NY, USA) and MedCalc Statistical Software version 22.023 (MedCalc Software Ltd., Ostend, Belgium). The reproducible Firth reanalysis and supplementary analyses were performed using Python version 3.12.14 and SciPy version 1.17.0. All tests were two-sided, and *p* < 0.05 was considered statistically significant. Multiple exploratory comparisons were not adjusted for multiplicity.

## 3. Results

### 3.1. Baseline Characteristics of the Study Population

The analytical sample comprised 357 patients with complete contemporaneous treatment and ocular-surface data. Their median age was 64 years (IQR 55–74), and 189 (52.9%) were women. Documented DED was present in 171 patients (47.9%). This proportion describes the selected sample of patients undergoing ocular-surface assessment with complete records and should not be interpreted as the prevalence among all glaucoma patients treated at the center. Primary open-angle glaucoma was the most common subtype (250/357, 70.0%). A preserved-exposure regimen was recorded for 266 patients (74.5%), whereas 91 (25.5%) received an exclusively PF regimen. Baseline characteristics are summarized in Table 1.

A total of 357 patients met the predefined eligibility criteria and were included in the final analysis. Because the retrospective analytical dataset did not retain a complete screening log with counts for each exclusion reason, a detailed numerical participant-flow breakdown could not be reconstructed.

### 3.2. Comparison Between Patients with and Without Dry Eye Disease

Patients with documented DED were older and had a longer duration of topical treatment than patients without documented DED. They also received a greater number of active IOP-lowering ingredients (all *p* < 0.001). Patients with documented DED had shorter TBUT, lower Schirmer test values, and higher OSDI scores than patients without documented DED (all *p* < 0.001). However, because TBUT, Schirmer test values, and OSDI scores may have contributed to the treating ophthalmologist’s diagnosis, these between-group differences are subject to potential incorporation bias and do not represent independent confirmation of DED status. Significant differences between the groups were observed across glaucoma subtypes (overall *p* = 0.024), primarily because normal-tension glaucoma was less frequent among patients with documented DED. Sex was not associated with DED status (*p* = 0.336).

The use of beta-blockers, alpha-agonists, carbonic anhydrase inhibitors, and fixed-combination therapy was more frequent among patients with documented DED (all *p* < 0.001). Prostaglandin analogue use also differed between groups, although the absolute difference was small (99.4% vs. 95.7%; *p* = 0.038). Artificial-tear use, diabetes mellitus, hypertension, and previous oculoplastic surgery were more frequent among patients with documented DED. All 12 patients with previous oculoplastic surgery had documented DED.

Documented DED occurred in 1/91 patients receiving an exclusively PF regimen (1.1%; 95% CI 0.2–6.0%) versus 170/266 receiving a preserved-exposure regimen (63.9%; 95% CI 58.0–69.4%; *p* < 0.001). These percentages use the treatment-group denominators. The single PF event and limited overlap between treatment groups constrain interpretation. The PF association was also examined using Firth penalization. Detailed comparisons according to documented DED status are presented in Table 2, and principal DED proportions with confidence intervals are presented in Supplementary Table S6.

Patient and treatment characteristics stratified by preservative exposure status are presented in Supplementary Table S3. Patients receiving PF regimens exclusively were younger and had shorter documented treatment duration than those receiving preserved-exposure regimens (median age, 57 vs. 66 years; median duration, 28 vs. 88.5 months). All 91 patients receiving PF regimens exclusively used one active ingredient, and none received fixed-combination therapy. In the preserved-exposure group, 30/266 patients used one ingredient and 107/266 received fixed-combination therapy. Cross-tabulations of preservative exposure, ingredient count, fixed-combination use, and documented DED demonstrate limited overlap between treatment groups.

### 3.3. Active-Ingredient Burden and Treatment Duration

Documented DED was more prevalent among patients receiving at least two active IOP-lowering ingredients (143/236; 60.6%; 95% CI 54.2–66.6%) than among those receiving one ingredient (28/121; 23.1%; 95% CI 16.5–31.4%; *p* < 0.001). Patients receiving multiple ingredients also had shorter TBUT, lower Schirmer test values, and higher OSDI scores (all *p* < 0.001). The corresponding descriptive ocular-surface measures are shown in Table 3. Documented treatment duration was analyzed continuously in Model 2.

Among the 121 patients receiving one active IOP-lowering ingredient, 28 had documented DED, compared with 143 of 236 patients receiving at least two active IOP-lowering ingredients. An exclusively PF regimen was more frequent among patients receiving one active IOP-lowering ingredient, whereas fixed-combination therapy and artificial-tear use were more frequent among patients receiving multiple active IOP-lowering ingredients. These differences indicate that active-ingredient count was closely related to treatment composition and should not be interpreted as an isolated measure of preservative exposure.

The previous comparison using a 12-month treatment-duration cutoff was removed. No documented clinical or empirical justification for the cutoff was available, and it divided the sample into 32 versus 325 patients. Continuous duration estimates and functional-form sensitivity analyses are presented in Table 4 and Supplementary Table S5.

### 3.4. Multivariable Logistic Regression

Treatment duration and active-ingredient count were strongly correlated (Spearman ρ = 0.860; *p* < 0.001) and were evaluated in complementary models. In Model 1, each additional active IOP-lowering ingredient was associated with higher odds of documented DED after adjustment for age, sex, and glaucoma subtype (aOR 2.65, 95% CI 1.88–3.74; *p* < 0.001). In Model 2, longer treatment duration was associated with higher DED odds (aOR 1.12 per 12 months, 95% CI 1.05–1.20; *p* < 0.001). Full model estimates are shown in Table 4.

In the extended conventional model, active-ingredient count was no longer independently associated with documented DED (aOR 1.02, 95% CI 0.69–1.51; *p* = 0.916), whereas fixed-combination therapy was associated with higher odds (aOR 3.23, 95% CI 1.74–5.98; *p* < 0.001). An exclusively PF regimen was associated with markedly lower odds (aOR 0.011, 95% CI 0.001–0.084; *p* < 0.001). However, only one of 91 patients receiving an exclusively PF regimen had documented DED, compared with 170 of 266 patients receiving a preserved-exposure regimen. Firth penalization yielded a similarly large association (penalized aOR 0.017, 95% CI 0.003–0.091; *p* < 0.001), although the PF comparison remained limited by the single event in that group. The exclusion analysis is reported in Supplementary Table S8. Extended model estimates are shown in Table 5.

Treatment coefficients in the extended model represent conditional associations adjusted for the other included covariates. The active-ingredient coefficient represents the association per additional ingredient, assuming a linear relationship with the log odds of documented DED. The fixed-combination coefficient compares regimens with and without fixed-combination use at the same modeled ingredient count and other covariate values. The PF coefficient compares PF and preserved-exposure regimens exclusively at the same modeled covariate values, subject to the limited overlap between treatment groups. These coefficients should not be interpreted as independent causal effects.

PF and preserved-exposure regimens overlapped in ingredient count only among patients receiving one active ingredient without fixed-combination therapy. Documented DED occurred in 1/91 PF patients and 27/30 preserved-exposure patients in this stratum. No PF patients received multiple ingredients or fixed combinations. Thus, the adjusted PF coefficient should not be interpreted as a comparison supported across all treatment-intensity strata.

The association between active-ingredient count and documented DED was substantially attenuated after exclusively PF regimen status, fixed-combination therapy, hypertension, and diabetes mellitus were added to the model. Active-ingredient count was no longer independently associated with DED in either the conventional extended model or the corresponding Firth model. Fixed-combination therapy remained associated with approximately three-fold higher DED odds in both analyses. Because these treatment variables capture overlapping aspects of treatment intensity and regimen composition, the extended model was considered exploratory.

Among 236 patients receiving at least two active ingredients, 107 received a fixed combination and 129 did not. Documented DED occurred in 84/107 (78.5%) and 59/129 (45.7%), respectively. Patient and treatment characteristics, ingredient-stratified DED counts, and an exploratory supplementary model are presented in Supplementary Table S4. All patients in this subset received preserved-exposure regimens. Fixed-combination use remained associated with documented DED after adjustment for categorical ingredient count, age, sex, glaucoma subtype, hypertension, and diabetes mellitus (aOR 4.33, 95% CI 2.28–8.23; *p* < 0.001). This analysis was not adjusted for treatment duration, drug-class composition, or glaucoma severity and therefore does not establish an independent formulation-specific effect.

Patient and documented DED counts across ingredient-count categories are presented in Supplementary Table S5. Modeling ingredient count categorically did not clearly improve fit over the linear specification in Model 1 (likelihood-ratio *p* = 0.075), but improved fit in the conventional extended model (*p* < 0.001). Thus, the extended-model estimates were sensitive to the functional form of ingredient count, and the null per-ingredient coefficient should not be generalized to the categorical specification. For treatment duration in Model 2, the restricted cubic spline analysis did not provide clear evidence of departure from linearity (*p* = 0.394).

Hypertension reached statistical significance in the conventional linear extended model but was marginally non-significant after Firth penalization. Diabetes mellitus, age, sex, and glaucoma subtype were not independently associated with documented DED in these models. After exclusion of the 12 patients with previous oculoplastic surgery, 345 patients and 159 DED events remained. In the conventional model, the aORs were 1.00 (95% CI 0.66–1.51; *p* = 0.994) for ingredient count, 0.011 (95% CI 0.001–0.087; *p* < 0.001) for an exclusively PF regimen, and 3.57 (95% CI 1.90–6.71; *p* < 0.001) for fixed-combination therapy. Corresponding Firth estimates were 1.00 (95% CI 0.66–1.50; *p* = 0.996), 0.018 (95% CI 0.003–0.095; *p* < 0.001), and 3.38 (95% CI 1.81–6.29; *p* < 0.001), respectively. Full exclusion-model estimates are provided in Supplementary Table S8.

### 3.5. Correlation Analyses

All six treatment–ocular-surface correlations are presented together in Supplementary Table S7. Active-ingredient count correlated negatively with TBUT (Spearman ρ = −0.904) and Schirmer values (ρ = −0.858), and positively with OSDI (ρ = 0.843). Documented treatment duration correlated negatively with TBUT (ρ = −0.751) and Schirmer values (ρ = −0.708), and positively with OSDI (ρ = 0.691); all *p* < 0.001, *n* = 357. Value-frequency distributions are provided in Supplementary Table S2. Among the 121 patients receiving one active ingredient, 72 (59.5%) had the joint pattern of TBUT = 10 s, Schirmer = 15 mm, and OSDI = 20. Recalculation reproduced the originally reported four correlations and added the two omitted combinations. The origin of the clustering could not be established from the analytical dataset; integer recording alone does not adequately explain the pattern across all three measures. These correlations should therefore be interpreted cautiously.

## 4. Discussion

In this retrospective cross-sectional study, almost half of patients receiving topical antiglaucoma therapy had documented DED. Greater active-ingredient burden and longer treatment duration were associated with documented DED in complementary models, but their independent contributions could not be fully separated because they were strongly correlated. An exclusively PF regimen was associated with markedly lower DED odds, whereas fixed-combination therapy was associated with higher odds in the extended model. However, active-ingredient count was no longer independently associated with DED after treatment formulation and comorbidities were added, indicating that active-ingredient count, formulation, and regimen composition captured overlapping dimensions of treatment burden.

The observed DED prevalence of 47.9% lies within the wide range reported in treated glaucoma populations. Küçük et al. reported a prevalence of 27% in a retrospective study that used documented dry eye and artificial-tear use, whereas Palikhey et al. reported a substantially higher prevalence using an OSDI-based definition [9,14]. Studies combining symptom-based and objective ocular-surface assessments have likewise reported heterogeneous estimates [6,7,15,16]. In the present study, DED was based on the treating ophthalmologist’s documented diagnosis rather than a retrospectively imposed uniform algorithm. Because TBUT, Schirmer test values, and OSDI scores may have contributed to the treating ophthalmologist’s documented DED diagnosis, differences in these measures between the DED and non-DED groups are subject to potential incorporation bias and should be interpreted as descriptive rather than as independent validation of DED status.

The association between treatment burden and ocular-surface dysfunction is consistent with previous glaucoma studies [6,7,8,12]. Potential mechanisms include exposure to preservatives and active compounds. BAK can disrupt epithelial integrity, promote oxidative stress and inflammatory signaling, reduce goblet-cell viability, and impair tear-film stability [2,10,17,18,19]. Active agents may also contribute to ocular-surface dysfunction, and greater active-ingredient count may reflect more frequent instillation and more complex regimens [2,20]. Nevertheless, the present dataset did not quantify BAK concentration, daily drop frequency, or cumulative preservative exposure. Active-ingredient count should therefore not be interpreted as a direct measure of preservative dose.

Recent clinical studies provide further support for an association between topical antiglaucoma treatment and ocular-surface abnormalities, while also illustrating the importance of treatment formulation. Srivastava et al. found less favorable symptom scores and ocular-surface parameters among patients receiving topical antiglaucoma medications than among treatment-naïve patients with primary open-angle glaucoma [21]. In the 2024 PRAMOS study, ocular-surface signs were also more frequent among patients receiving preserved prostaglandin analogue monotherapy than among those receiving preservative-free formulations [22]. Although these findings are consistent with the present results, differences in study populations, treatment regimens, outcome definitions, and exposure duration limit direct quantitative comparison.

Active-ingredient count and treatment duration were strongly correlated and were therefore evaluated in separate primary models. Each was associated with documented DED after adjustment for age, sex, and glaucoma subtype. However, active-ingredient count was no longer associated with documented DED in the extended model incorporating preservative exposure status, fixed-combination therapy, hypertension, and diabetes mellitus (aOR 1.02, 95% CI 0.69–1.51; *p* = 0.916). The null per-ingredient association applies to the linear specification of the extended model. A categorical specification significantly improved model fit, indicating that interpretation of ingredient count was sensitive to functional form. Category-specific estimates were constrained by limited overlap between treatment groups and sparse outcomes, particularly the presence of only one patient without documented DED in the four-ingredient group. These estimates should not be interpreted as evidence of protective or harmful effects of particular ingredient counts.

Fixed-combination therapy remained associated with higher odds of documented DED in both the conventional and Firth models. In the supplementary analysis restricted to patients receiving at least two active ingredients, this association persisted after adjustment for ingredient-count categories, age, sex, glaucoma subtype, hypertension, and diabetes mellitus (aOR 4.33, 95% CI 2.28–8.23; *p* < 0.001; Supplementary Table S4). All patients in this subset received preserved-exposure regimens. However, fixed-combination use may identify patients requiring more intensive IOP-lowering treatment or those with more advanced glaucoma and a history of treatment escalation. The supplementary comparison did not match specific active ingredients or adjust for treatment duration or glaucoma severity. Current ingredient count also does not capture previous multidrug exposure, dosing frequency, or cumulative treatment intensity. Accordingly, residual confounding by indication remains possible, and the observed association should not be interpreted as an independent detrimental effect of fixed-combination formulations themselves. Firth penalization addresses sparse-data estimation but does not resolve unmeasured confounding.

An exclusively PF regimen showed an extremely large inverse association with documented DED, but only one DED event occurred among 91 patients receiving PF regimens exclusively. Conventional and Firth-penalized models gave similar estimates, yet penalization cannot eliminate treatment-selection bias or compensate for the limited overlap between exposure groups. Treatment switching, previous exposure to preserved formulations, clinician prescribing preferences, disease severity, and unmeasured ocular-surface history were unavailable. The result should therefore be interpreted as an association requiring prospective confirmation, not as evidence of a protective causal effect.

The PF-stratified descriptions revealed substantial differences in treatment composition and documented duration. All exclusively PF patients received one active ingredient, whereas most preserved-exposure patients received multiple ingredients. No PF patients received fixed combinations. This limited covariate overlap restricts interpretation of the adjusted PF estimate, particularly its generalization to multidrug regimens. Firth penalization addresses sparse-data estimation but cannot create comparable treatment groups or eliminate treatment-selection bias and residual confounding.

Experimental evidence and clinical formulation-comparison studies provide a rationale for examining ocular-surface tolerability [11,12,19]. Oddone et al. reported improved ocular-surface signs and symptoms after switching patients from preserved latanoprost to a PF tafluprost–timolol fixed combination [23]. This switch simultaneously removed preservatives, changed the prostaglandin analogue, added timolol, and introduced a fixed combination; its effects cannot be attributed solely to preservative removal. Baudouin et al. compared a PF latanoprost cationic emulsion with preserved latanoprost solution and reported non-inferior IOP lowering and greater improvement in corneal fluorescein staining over 12 weeks [24]. Although both treatments contained latanoprost, the comparison also changed the vehicle and formulation, so it does not isolate the effect of preservative absence. These comparability limitations are specified in Supplementary Table S1 and prevent direct causal extrapolation to the present observational PF association.

Hypertension reached nominal significance in the conventional extended model but was borderline after Firth penalization. Because individual antihypertensive agents were unavailable, this result may reflect residual confounding and should be considered exploratory. Diabetes was associated with DED in unadjusted analysis but not after multivariable adjustment. More detailed prospective studies are needed to distinguish systemic disease effects from medication-related effects [4,20].

These findings support incorporating ocular-surface assessment into long-term glaucoma care, particularly for patients using complex multidrug regimens. Treatment review may include symptoms, adherence, instillation frequency, preserved versus PF formulations, and opportunities to simplify therapy while maintaining adequate intraocular pressure control. The present results do not establish that switching to an exclusively PF regimen will prevent or reverse DED, but they provide a rationale for prospective treatment-switching studies with standardized ocular-surface outcomes.

This study has several limitations. Its retrospective cross-sectional design precludes causal or temporal inference, and baseline ocular-surface status and treatment-switching history were unavailable. The analytical dataset did not distinguish treatment used before the index examination from prescriptions issued afterward. Same-visit treatment changes prompted by ocular-surface findings may therefore have affected exposure classification. DED was based on a routinely documented diagnosis; a uniform prespecified diagnostic algorithm and standardized testing sequence could not be verified. TBUT and Schirmer values lacked ocular laterality and an eye-selection or aggregation rule. TBUT technique, Schirmer duration and anesthesia use, and OSDI version and original scoring procedures were not independently verified. These gaps limit measurement comparability and assessment of inter-eye differences. The single-center tertiary-care setting may limit generalizability. Oculoplastic procedure type and timing were unavailable, although excluding the 12 affected patients did not materially alter the PF estimate. Pronounced clustering was observed across TBUT, Schirmer test values, and OSDI scores. A source-record check was not performed; therefore, we could not determine whether the repeated values represented contemporaneous measurements, default entries, recording ceilings, categorical recording, or carried-forward results. This unresolved issue limits interpretation of the ocular-surface comparisons and correlations. Detailed dosing frequency, cumulative preservative exposure, systemic medication classes, corneal staining, osmolarity, and meibomian gland assessments were unavailable. Ocular-surface testing was prompted by symptoms or clinical judgment rather than performed systematically in all glaucoma patients. Restricting inclusion to patients with complete contemporaneous ocular-surface assessments may therefore have introduced selection bias and affected both the observed proportion of documented DED and treatment-related associations. The reported prevalence applies only to this selected analytical sample. Because TBUT, Schirmer testing, and OSDI may have contributed to the treating ophthalmologist’s clinical diagnosis, comparisons of these measures between DED and non-DED groups are subject to potential incorporation bias. Glaucoma severity, visual-field status, target IOP, and prior treatment escalation were unavailable and may have contributed to confounding by indication, particularly for active-ingredient burden and fixed-combination therapy. In addition, incomplete information on treatment received before presentation to our institution may have resulted in misclassification of cumulative treatment duration. The Firth confidence intervals and tests used Wald approximations, which may be less reliable with sparse events than profile-penalized likelihood inference. Finally, multiple exploratory comparisons were performed without multiplicity adjustment, and the exclusively PF group contained only one DED event. Strengths include the relatively large patient-level sample, availability of treatment and ocular-surface entries from the selected encounter, transparent separation of highly correlated predictors, and penalized sensitivity analysis for sparse exposure-outcome data.

## 5. Conclusions

Documented DED was present in 47.9% of this selected cohort with complete treatment and ocular-surface data. Active-ingredient count and documented treatment duration were associated with DED in separate primary adjusted models. Active-ingredient count was not associated with DED in the linear extended model, but categorical modeling significantly improved model fit, indicating sensitivity to functional form. An exclusively preservative-free regimen was associated with lower DED odds and fixed-combination therapy with higher odds. However, treatment selection, limited overlap between treatment groups, residual confounding, and the single DED event in the exclusively preservative-free group constrain interpretation. These findings support attention to ocular-surface health during glaucoma care but do not establish causal effects of treatment characteristics. Prospective studies with standardized diagnostic criteria and detailed treatment histories are needed to clarify these associations.

## Figures and Tables

**Table 1:** Baseline characteristics of the study population.

Variable	Total Cohort (*n* = 357)
Age, years	64 (55–74)
Male sex, *n* (%)	168 (47.1)
Female sex, *n* (%)	189 (52.9)
Dry eye disease, *n* (%)	171 (47.9)
Primary open-angle glaucoma, *n* (%)	250 (70.0)
Pseudoexfoliative glaucoma, *n* (%)	61 (17.1)
Normal-tension glaucoma, *n* (%)	29 (8.1)
Other glaucoma subtype, *n* (%)	17 (4.8)
Treatment duration, months	65 (31–114)
Number of active IOP-lowering ingredients	2 (1–3)
Prostaglandin analogue	348 (97.5)
Beta-blocker	245 (68.6)
Alpha-agonist	99 (27.7)
Carbonic anhydrase inhibitor	33 (9.2)
Fixed-combination therapy	107 (30.0)
Exclusively preservative-free regimen	91 (25.5)
Artificial tears	162 (45.4)
Diabetes mellitus	76 (21.3)
Hypertension	199 (55.7)
Previous oculoplastic surgery	12 (3.4)
TBUT, s	8 (5–10)
Schirmer test, mm	10 (7–15)
OSDI score	40 (20–50)

Data are median (IQR) or *n* (%). DED, dry eye disease; OSDI, Ocular Surface Disease Index; TBUT, tear film breakup time. Drug-class counts are not mutually exclusive and include patients with recorded class indicators within fixed-combination regimens. Fixed-combination status is reported separately. The 95% Wilson CI for the overall DED proportion is 42.8–53.1%.

**Table 2:** Comparison according to documented dry eye disease status.

Variable	DED (*n* = 171)	Non-DED (*n* = 186)	*p*-Value
Age, years	68.0 (59.0–78.5)	60.5 (52.0–68.0)	<0.001
Treatment duration, months	94.0 (51.0–139.0)	47.5 (28.0–87.0)	<0.001
Number of active IOP-lowering ingredients	2.0 (2.0–3.0)	1.5 (1.0–2.0)	<0.001
TBUT, s	6.0 (4.0–7.0)	9.0 (8.0–10.0)	<0.001
Schirmer test, mm	7.0 (5.0–9.0)	14.5 (12.0–15.0)	<0.001
OSDI score	50.0 (45.0–75.0)	25.0 (20.0–30.0)	<0.001
Female sex	86 (50.3)	103 (55.4)	0.336
Primary open-angle glaucoma	125 (73.1)	125 (67.2)	0.024 *
Pseudoexfoliative glaucoma	31 (18.1)	30 (16.1)	
Normal-tension glaucoma	6 (3.5)	23 (12.4)	
Other glaucoma subtype	9 (5.3)	8 (4.3)	
Prostaglandin analogue	170 (99.4)	178 (95.7)	0.038
Beta-blocker	144 (84.2)	101 (54.3)	<0.001
Alpha-agonist	75 (43.9)	24 (12.9)	<0.001
Carbonic anhydrase inhibitor	31 (18.1)	2 (1.1)	<0.001
Fixed-combination therapy	84 (49.1)	23 (12.4)	<0.001
Exclusively preservative-free regimen	1 (0.6)	90 (48.4)	<0.001
Artificial tears	103 (60.2)	59 (31.7)	<0.001
Diabetes mellitus	48 (28.1)	28 (15.1)	0.003
Hypertension	109 (63.7)	90 (48.4)	0.004
Previous oculoplastic surgery	12 (7.0)	0 (0.0)	<0.001

Data are median (IQR) or *n* (%). Non-DED denotes no documented DED diagnosis at the index visit and does not necessarily indicate absence of disease. * Ocular-surface measures are presented descriptively. Because TBUT, Schirmer test values, and OSDI scores may have informed the treating ophthalmologist’s documented DED diagnosis, between-group differences are subject to potential incorporation bias and should not be interpreted as independent validation of DED status. Treatment percentages in this table use the outcome-group denominators; they do not represent DED proportions within treatment groups. The direct PF comparison is 1/91 (1.1%; 95% CI 0.2–6.0%) versus 170/266 (63.9%; 95% CI 58.0–69.4%). Drug-class counts are not mutually exclusive and overlap with fixed-combination use.

**Table 3:** Documented DED and ocular-surface measures by active-ingredient burden.

Variable	1 Active Ingredient	≥2 Active Ingredients
*n*	121	236
Documented DED, *n* (%)	28 (23.1%; 95% CI 16.5–31.4%)	143 (60.6%; 95% CI 54.2–66.6%)
TBUT, s	10.0 (10.0–10.0)	6.0 (4.0–8.0)
Schirmer test, mm	15.0 (15.0–15.0)	8.0 (5.0–12.0)
OSDI score	20.0 (20.0–20.0)	45.0 (30.0–75.0)

Data are median (IQR) unless stated otherwise. DED cells present *n* (%; 95% Wilson CI). Between-group *p* < 0.001 for ocular-surface comparisons and documented DED. Ocular-surface differences are descriptive and subject to the measurement limitations described in the Methods and Discussion.

**Table 4:** Complementary multivariable models for documented DED.

Variable	Model 1 aOR (95% CI)	*p*	Model 2 aOR (95% CI)	*p*
Age, per year	1.01 (0.99–1.04)	0.333	1.03 (1.01–1.06)	0.006
Female vs. male	0.84 (0.53–1.34)	0.471	0.88 (0.56–1.39)	0.596
Pseudoexfoliative vs. POAG	0.93 (0.50–1.72)	0.819	1.23 (0.67–2.26)	0.499
Normal-tension vs. POAG	0.43 (0.16–1.14)	0.091	0.35 (0.13–0.92)	0.033
Other subtype vs. POAG	0.99 (0.34–2.93)	0.992	1.21 (0.42–3.45)	0.722
Active-ingredient count, per active ingredient	2.65 (1.88–3.74)	<0.001	—	—
Treatment duration, per 12 months	—	—	1.12 (1.05–1.20)	<0.001

Both models included 357 patients and 171 DED events. Model 1 included active-ingredient count but not treatment duration; Model 2 included treatment duration but not active-ingredient count. POAG, primary open-angle glaucoma. The duration estimate per 12 months is a rescaling of the same fitted linear coefficient, not a separate model.

**Table 5:** Extended conventional and Firth-penalized logistic regression models.

Variable	Conventional aOR (95% CI)	*p*	Firth aOR (95% CI)	*p*
Age, per year	1.01 (0.98–1.04)	0.599	1.01 (0.98–1.04)	0.608
Female vs. male	0.85 (0.50–1.44)	0.545	0.86 (0.51–1.45)	0.561
Pseudoexfoliative vs. POAG	0.54 (0.26–1.09)	0.087	0.55 (0.27–1.11)	0.095
Normal-tension vs. POAG	0.39 (0.12–1.21)	0.103	0.41 (0.13–1.26)	0.119
Other subtype vs. POAG	0.81 (0.25–2.62)	0.719	0.80 (0.25–2.56)	0.702
Active-ingredient count, per active ingredient	1.02 (0.69–1.51)	0.916	1.02 (0.69–1.51)	0.917
Exclusively PF vs. preserved-exposure regimen	0.011 (0.001–0.084)	<0.001	0.017 (0.003–0.091)	<0.001
Fixed-combination therapy	3.23 (1.74–5.98)	<0.001	3.07 (1.67–5.64)	<0.001
Hypertension	1.76 (1.01–3.05)	0.046	1.72 (1.00–2.97)	0.052
Diabetes mellitus	1.65 (0.85–3.18)	0.139	1.60 (0.83–3.06)	0.158

Both models included 357 patients and 171 DED events. The same covariates were used in both models. PF, preservative-free; POAG, primary open-angle glaucoma. Firth estimates were reproduced for this revision using Supplementary Code 1. Both columns use Wald inference; Firth standard errors use the ordinary expected information evaluated at the penalized estimates, not profile-penalized likelihood inference.

## Data Availability

The data are not publicly available because they contain clinical information subject to privacy and institutional restrictions. Deidentified data may be available from the corresponding author upon request and with approval from the relevant institution and ethics committee.

## Supplementary Materials

The following supporting information can be downloaded at: https://www.mdpi.com/article/10.3390/biomedicines14102344/s1, Supplementary Table S1: Selected studies evaluating ocular-surface disease or tolerability in patients receiving topical antiglaucoma therapy. Supplementary Table S2: Value-frequency distributions of TBUT, Schirmer test values, and OSDI scores overall and by active-ingredient count. Supplementary Table S3: Patient and treatment characteristics by preservative exposure status and cross-tabulations of treatment variables with documented DED. Supplementary Table S4: Patient and treatment characteristics by fixed-combination use among patients receiving at least two active IOP-lowering ingredients, ingredient-stratified documented DED counts, and an exploratory adjusted analysis. Supplementary Table S5: Patient and documented DED counts by active-ingredient count and sensitivity analyses of the functional form of ingredient count and treatment duration. Supplementary Table S6: Principal documented DED proportions with Wilson 95% confidence intervals. Supplementary Table S7: All six correlations between treatment variables and ocular-surface measures. Supplementary Table S8: Conventional and Firth models after exclusion of patients with previous oculoplastic surgery. Supplementary Code 1: Reproducible Firth implementation and the additional proportion, correlation, and exclusion analyses.

## Abbreviations

The following abbreviations are used in this manuscript:

aOR	Adjusted odds ratio
BAK	Benzalkonium chloride
CI	Confidence interval
DED	Dry eye disease
IQR	Interquartile range
IOP	Intraocular pressure
OSD	Ocular surface disease
OSDI	Ocular Surface Disease Index
PF	Preservative-free
POAG	Primary open-angle glaucoma
STROBE	Strengthening the Reporting of Observational Studies in Epidemiology
TBUT	Tear film breakup time
