# Supplementary material for: Documented Dry Eye Disease in Patients Receiving Topical Antiglaucoma Therapy: Associations with Treatment Characteristics
Source: Biomedicines. 2026 Oct 15;14(10):2344. doi: 10.3390/biomedicines14102344 (PMC13702091; doi:10.3390/biomedicines14102344)
Supplement: Supplementary file 1 [file biomedicines-14-02344-s001.zip › Supplementary_Table_S1.pdf]

Supplementary Table S1. Selected studies evaluating ocular-surface disease or tolerability in patients receiving topical antiglaucoma therapy.

Study [main-text ref.]	Design and setting	Sample	Treatment exposure/comparator	Ocular-surface assessment	Principal findings	Key comparability limitation
Ramli et al., 2015 [7]	Cross-sectional case-comparison study; Malaysia	105 treated glaucoma patients; 102 untreated controls	Topical antiglaucoma treatment; number of medications; BAK-containing and PF drops	TBUT, corneal staining, Schirmer test, OSDI	OSD prevalence ranged from 37% to 91% depending on the test. Abnormal TBUT increased with a greater number of topical medications. BAK exposure was associated with approximately threefold higher odds of abnormal OSDI.	Different tests produced markedly different prevalence estimates; PF exposure was not equivalent to an exclusively PF multidrug regimen.
Saade et al., 2015 [8]	Prospective, controlled cross-sectional study; single US center	31 treated POAG patients; 30 controls	At least one topical IOP-lowering medication; treatment intensity index (drops/week × years)	OSDI, TBUT, corneal and conjunctival lissamine-green staining; right eye selected	Treated patients had higher OSDI scores and more abnormal TBUT and staining. OSDI correlated with treatment intensity ($r = 0.46$).	Small sample; selected one eye; excluded patients with prior OSD diagnosis or treatment.
Kobia-Acquah et al., 2021 [6]	Hospital-based descriptive cross-sectional study; Ghana	100 treated glaucoma patients	Topical therapy for ≥6 months; number and duration of medications; preservative type	OSDI, Schirmer test, TBUT	DED prevalence was 23%. Use of >2 medications, treatment for ≥5 years, older age, and combined preservative exposure were associated with higher DED odds.	DED definition and exposure classification differ from the present study; modest sample size.
Palikhey et al., 2022 [14]	Descriptive cross-sectional study; tertiary center, Nepal	250 glaucoma patients receiving topical agents	Topical antiglaucoma therapy	OSDI-based symptom assessment	A high burden of symptomatic DED was reported using an OSDI-based definition.	Primarily symptom-defined DED; no directly comparable clinician-documented diagnosis or standardized objective-test algorithm.
Küçük et al., 2024 [9]	Retrospective clinical-record study	1274 patients receiving topical antiglaucoma medication	Routine topical antiglaucoma treatment	Documented dry eye and/or artificial-tear use in routine records	Documented dry eye/artificial-tear use was identified in 27% of patients.	Record-based outcome may underestimate DED and differs from prospective symptom/sign testing.

Study [main-text ref.]	Design and setting	Sample	Treatment exposure/comparator	Ocular-surface assessment	Principal findings	Key comparability limitation
Bastelica et al. (PRAMOS), 2024 [22]	Multicenter real-world observational study; France	344 patients/eyes on prostaglandin monotherapy (PF n = 271; preserved n = 73)	PF versus preserved prostaglandin-analogue monotherapy	Symptoms, TBUT, conjunctival hyperemia, corneal/conjunctival staining	At least one symptom or sign was present in 79.4%. Preserved therapy was associated with more conjunctival hyperemia and greater corneal staining; TBUT and most symptoms were similar between formulation groups.	Monotherapy population; nonrandomized treatment selection; preserved and PF groups differed in treatment duration.
Oddone et al., 2024 [23]	Prospective interventional switching study; Italy	43 OAG/OHT patients	Switch from preserved latanoprost to PF tafluprost–timolol fixed combination	24-hour efficacy and tolerability; ocular-surface signs and symptoms	Switching maintained IOP control and improved ocular-surface tolerability measures.	Single-arm switching study without a concurrent control. The switch removed preservatives, changed latanoprost to tafluprost, added timolol, and introduced a fixed combination. Improvements cannot be attributed solely to preservative removal; short follow-up and selected population also limit comparability.
Baudouin et al., 2025 [24]	Randomized phase III non-inferiority trial; multicenter	386 OAG/OHT patients (PF n = 193; preserved n = 193)	PF latanoprost emulsion versus preserved latanoprost for 12 weeks	Corneal fluorescein staining, ocular-surface symptom score, hyperemia and adverse events	PF latanoprost was non-inferior for IOP reduction and produced a larger improvement in corneal fluorescein staining; symptom-score difference was not statistically significant.	The same active drug was delivered as a PF cationic emulsion versus a preserved solution. Preservative status and vehicle/formulation changed together, so this comparison does not isolate preservative removal. Short

Study [main-text ref.]	Design and setting	Sample	Treatment exposure/comparator	Ocular-surface assessment	Principal findings	Key comparability limitation
						randomized follow-up and trial outcomes differ from routine clinician-documented DED.

Notes: This table presents a selected narrative comparison of studies cited in the main manuscript and is not a systematic review. Reference numbers correspond to the reference list of the main manuscript. Study definitions of DED/OSD and preservative exposure were heterogeneous; prevalence estimates and effect sizes should therefore not be compared directly.

Abbreviations: BAK, benzalkonium chloride; DED, dry eye disease; IOP, intraocular pressure; OAG, open-angle glaucoma; OHT, ocular hypertension; OSD, ocular surface disease; OSDI, Ocular Surface Disease Index; PF, preservative-free; POAG, primary open-angle glaucoma; TBUT, tear film breakup time.
