# Supplementary material for: Documented Dry Eye Disease in Patients Receiving Topical Antiglaucoma Therapy: Associations with Treatment Characteristics
Source: Biomedicines. 2026 Oct 15;14(10):2344. doi: 10.3390/biomedicines14102344 (PMC13702091; doi:10.3390/biomedicines14102344)
Supplement: Supplementary file 1 [file biomedicines-14-02344-s001.zip › Supplementary_Table_S2.pdf]

Supplementary Table S2

Value-frequency distributions of TBUT, Schirmer test values, and OSDI scores overall and by active-ingredient count.

A. TBUT (s)

Recorded value	Overall (n = 357)	1 ingredient (n = 121)	2 ingredients (n = 133)	3 ingredients (n = 72)	4 ingredients (n = 31)
1	7 (2.0)	0 (0.0)	0 (0.0)	0 (0.0)	7 (22.6)
2	21 (5.9)	0 (0.0)	0 (0.0)	0 (0.0)	21 (67.7)
3	14 (3.9)	1 (0.8)	0 (0.0)	11 (15.3)	2 (6.5)
4	38 (10.6)	0 (0.0)	0 (0.0)	37 (51.4)	1 (3.2)
5	14 (3.9)	0 (0.0)	0 (0.0)	14 (19.4)	0 (0.0)
6	44 (12.3)	0 (0.0)	35 (26.3)	9 (12.5)	0 (0.0)
7	32 (9.0)	0 (0.0)	32 (24.1)	0 (0.0)	0 (0.0)
8	56 (15.7)	17 (14.0)	39 (29.3)	0 (0.0)	0 (0.0)
9	40 (11.2)	12 (9.9)	27 (20.3)	1 (1.4)	0 (0.0)
10	72 (20.2)	72 (59.5)	0 (0.0)	0 (0.0)	0 (0.0)
11	19 (5.3)	19 (15.7)	0 (0.0)	0 (0.0)	0 (0.0)
Total	357 (100.0)	121 (100.0)	133 (100.0)	72 (100.0)	31 (100.0)

Data are n (%). Percentages are calculated within each column and may not sum to 100.0 because of rounding. No missing values were present for this measure.

Supplementary Table S2 (continued)

B. Schirmer test (mm)

Recorded value	Overall (n = 357)	1 ingredient (n = 121)	2 ingredients (n = 133)	3 ingredients (n = 72)	4 ingredients (n = 31)
0	6 (1.7)	0 (0.0)	0 (0.0)	0 (0.0)	6 (19.4)
2	24 (6.7)	0 (0.0)	0 (0.0)	0 (0.0)	24 (77.4)
3	11 (3.1)	0 (0.0)	0 (0.0)	11 (15.3)	0 (0.0)
5	37 (10.4)	0 (0.0)	0 (0.0)	37 (51.4)	0 (0.0)
7	34 (9.5)	0 (0.0)	33 (24.8)	0 (0.0)	1 (3.2)
8	15 (4.2)	0 (0.0)	0 (0.0)	15 (20.8)	0 (0.0)
9	32 (9.0)	0 (0.0)	32 (24.1)	0 (0.0)	0 (0.0)
10	27 (7.6)	18 (14.9)	0 (0.0)	9 (12.5)	0 (0.0)
12	49 (13.7)	10 (8.3)	39 (29.3)	0 (0.0)	0 (0.0)
14	29 (8.1)	0 (0.0)	29 (21.8)	0 (0.0)	0 (0.0)
15	73 (20.4)	73 (60.3)	0 (0.0)	0 (0.0)	0 (0.0)
17	20 (5.6)	20 (16.5)	0 (0.0)	0 (0.0)	0 (0.0)
Total	357 (100.0)	121 (100.0)	133 (100.0)	72 (100.0)	31 (100.0)

Data are n (%). Percentages are calculated within each column and may not sum to 100.0 because of rounding. No missing values were present for this measure.

Supplementary Table S2 (continued)

C. OSDI score

Recorded value	Overall (n = 357)	1 ingredient (n = 121)	2 ingredients (n = 133)	3 ingredients (n = 72)	4 ingredients (n = 31)
5	18 (5.0)	18 (14.9)	0 (0.0)	0 (0.0)	0 (0.0)
15	2 (0.6)	2 (1.7)	0 (0.0)	0 (0.0)	0 (0.0)
20	72 (20.2)	72 (59.5)	0 (0.0)	0 (0.0)	0 (0.0)
25	29 (8.1)	0 (0.0)	29 (21.8)	0 (0.0)	0 (0.0)
30	40 (11.2)	1 (0.8)	39 (29.3)	0 (0.0)	0 (0.0)
35	11 (3.1)	11 (9.1)	0 (0.0)	0 (0.0)	0 (0.0)
40	26 (7.3)	17 (14.0)	0 (0.0)	9 (12.5)	0 (0.0)
45	47 (13.2)	0 (0.0)	32 (24.1)	15 (20.8)	0 (0.0)
50	33 (9.2)	0 (0.0)	33 (24.8)	0 (0.0)	0 (0.0)
70	1 (0.3)	0 (0.0)	0 (0.0)	0 (0.0)	1 (3.2)
75	37 (10.4)	0 (0.0)	0 (0.0)	37 (51.4)	0 (0.0)
80	11 (3.1)	0 (0.0)	0 (0.0)	11 (15.3)	0 (0.0)
90	24 (6.7)	0 (0.0)	0 (0.0)	0 (0.0)	24 (77.4)
95	6 (1.7)	0 (0.0)	0 (0.0)	0 (0.0)	6 (19.4)
Total	357 (100.0)	121 (100.0)	133 (100.0)	72 (100.0)	31 (100.0)

Data are n (%). Percentages are calculated within each column and may not sum to 100.0 because of rounding. No missing values were present for this measure.

TBUT, tear film breakup time; OSDI, Ocular Surface Disease Index. Counts reflect the supplied analytical dataset; these distributions do not establish the origin of repeated values or constitute verification against source records.
