# Supplementary material for: Documented Dry Eye Disease in Patients Receiving Topical Antiglaucoma Therapy: Associations with Treatment Characteristics
Source: Biomedicines. 2026 Oct 15;14(10):2344. doi: 10.3390/biomedicines14102344 (PMC13702091; doi:10.3390/biomedicines14102344)
Supplement: Supplementary file 1 [file biomedicines-14-02344-s001.zip › Supplementary_Table_S3.pdf]

Supplementary Table S3

Patient and treatment characteristics by preservative exposure status and cross-tabulations of treatment variables with documented DED.

**Characteristic**	**Exclusively PF (n = 91)**	**Preserved-exposure (n = 266)**
Age, years	57.0 (47.5–64.0)	66.0 (58.0–76.0)
Documented treatment duration, months	28.0 (12.0–38.0)	88.5 (53.0–127.8)
Active-ingredient count	1.0 (1.0–1.0)	2.0 (2.0–3.0)
Female sex	55 (60.4)	134 (50.4)
Primary open-angle glaucoma	63 (69.2)	187 (70.3)
Pseudoexfoliative glaucoma	11 (12.1)	50 (18.8)
Normal-tension glaucoma	15 (16.5)	14 (5.3)
Other glaucoma subtype	2 (2.2)	15 (5.6)
1 active ingredient	91 (100.0)	30 (11.3)
2 active ingredients	0 (0.0)	133 (50.0)
3 active ingredients	0 (0.0)	72 (27.1)
4 active ingredients	0 (0.0)	31 (11.7)
Fixed-combination therapy	0 (0.0)	107 (40.2)
Prostaglandin analogue	83 (91.2)	265 (99.6)
Beta-blocker	8 (8.8)	237 (89.1)
Alpha-agonist	0 (0.0)	99 (37.2)
Carbonic anhydrase inhibitor	0 (0.0)	33 (12.4)
Artificial-tear use	18 (19.8)	144 (54.1)
Diabetes mellitus	11 (12.1)	65 (24.4)
Hypertension	42 (46.2)	157 (59.0)
Previous oculoplastic surgery	0 (0.0)	12 (4.5)
Documented DED	1 (1.1)	170 (63.9)

Data are median (IQR) or n (%). Percentages use the treatment-group denominator. Glaucoma subtype and sex labels follow the manuscript coding; drug-class indicators are shown as supplied and are not mutually exclusive. IQRs use linear interpolation of sample quantiles.

Supplementary Table S3 (continued)

Cross-tabulation of preservative exposure, active-ingredient count, fixed-combination use, and documented DED

Regimen	Ingredients	Fixed combination	Patients, n	DED, n	Non-DED, n	DED, %
Exclusively PF	1	No	91	1	90	1.1
Exclusively PF	1	Yes	0	0	0	—
Exclusively PF	2	No	0	0	0	—
Exclusively PF	2	Yes	0	0	0	—
Exclusively PF	3	No	0	0	0	—
Exclusively PF	3	Yes	0	0	0	—
Exclusively PF	4	No	0	0	0	—
Exclusively PF	4	Yes	0	0	0	—
Preserved-exposure	1	No	30	27	3	90.0
Preserved-exposure	1	Yes	0	0	0	—
Preserved-exposure	2	No	81	32	49	39.5
Preserved-exposure	2	Yes	52	33	19	63.5
Preserved-exposure	3	No	36	16	20	44.4
Preserved-exposure	3	Yes	36	32	4	88.9
Preserved-exposure	4	No	12	11	1	91.7
Preserved-exposure	4	Yes	19	19	0	100.0

DED percentages use the number of patients in each row. Empty cells are retained to show lack of treatment overlap. Non-DED denotes absence of a documented DED diagnosis. PF, preservative-free; DED, dry eye disease; IQR, interquartile range.
