# Supplementary material for: Documented Dry Eye Disease in Patients Receiving Topical Antiglaucoma Therapy: Associations with Treatment Characteristics
Source: Biomedicines. 2026 Oct 15;14(10):2344. doi: 10.3390/biomedicines14102344 (PMC13702091; doi:10.3390/biomedicines14102344)
Supplement: Supplementary file 1 [file biomedicines-14-02344-s001.zip › Supplementary_Table_S4.pdf]

Supplementary Table S4

Patient and treatment characteristics by fixed-combination use among patients receiving at least two active IOP-lowering ingredients.

A Descriptive comparison

Characteristic	Without fixed combination (n = 129)	With fixed combination (n = 107)
Age, years	65 (57–75)	72 (62–80)
Documented treatment duration, months	91 (67–133)	101 (54–134)
Active-ingredient count	2 (2–3)	3 (2–3)
Sex code 1 (female in manuscript)	65 (50.4)	57 (53.3)
POAG (code 1)	92 (71.3)	67 (62.6)
Pseudoexfoliative glaucoma (code 2)	20 (15.5)	30 (28.0)
Normal-tension glaucoma (code 3)	10 (7.8)	3 (2.8)
Other glaucoma (code 4)	7 (5.4)	7 (6.5)
Two active ingredients	81 (62.8)	52 (48.6)
Three active ingredients	36 (27.9)	36 (33.6)
Four active ingredients	12 (9.3)	19 (17.8)
Prostaglandin analogue	129 (100.0)	107 (100.0)
Beta-blocker	129 (100.0)	107 (100.0)
Alpha-agonist	45 (34.9)	54 (50.5)
Carbonic anhydrase inhibitor	15 (11.6)	18 (16.8)
Exclusively preservative-free regimen	0 (0.0)	0 (0.0)
Artificial-tear use	73 (56.6)	59 (55.1)
Diabetes mellitus	29 (22.5)	30 (28.0)
Hypertension	76 (58.9)	64 (59.8)
Previous oculoplastic surgery	6 (4.7)	4 (3.7)
Documented DED	59 (45.7)	84 (78.5)

Data are median (IQR) or n (%), with within-group denominators. All 236 patients received preserved-exposure regimens. Drug-class indicators are reproduced as coded and overlap. Fixed-combination users may also receive additional formulations. Classification uses the database indicator; named prescriptions were not independently checked. No displayed values were missing. Sex and glaucoma labels follow manuscript coding.

Supplementary Table S4 continued

B Documented DED by ingredient count

Ingredient count	Without fixed combination: DED/n (%)	With fixed combination: DED/n (%)
2	32/81 (39.5%)	33/52 (63.5%)
3	16/36 (44.4%)	32/36 (88.9%)
4	11/12 (91.7%)	19/19 (100.0%)

Values are DED cases/total (%) within each cell. The four-ingredient group with fixed-combination use contains no non-DED patients, limiting interpretation of that stratum.

C Exploratory adjusted analysis

Covariate	Adjusted OR	95% CI	p value
Fixed-combination use	4.331	2.279–8.228	<0.001
Three vs two ingredients	1.799	0.834–3.881	0.134
Four vs two ingredients	25.399	3.093–208.564	0.003
Age, per 10 years	0.953	0.698–1.301	0.760
Sex code 1 vs 0	0.884	0.486–1.610	0.688
Glaucoma code 2 vs 1	0.810	0.380–1.727	0.585
Glaucoma code 3 vs 1	0.926	0.269–3.187	0.903
Glaucoma code 4 vs 1	0.858	0.235–3.127	0.816
Hypertension	1.865	1.000–3.479	0.050
Diabetes mellitus	1.259	0.608–2.607	0.534

Conventional maximum-likelihood logistic regression: n = 236, with 143 documented DED events and 93 patients without a documented DED diagnosis. Adjusted for categorical ingredient count, age, sex, glaucoma subtype, hypertension, and diabetes mellitus. Reference categories: no fixed combination, two ingredients, sex code 0, glaucoma code 1, no hypertension, and no diabetes. Age is expressed per 10 years. PF status was omitted because it was constant. Treatment duration was omitted, consistent with the original extended model; its Spearman correlation with ingredient count in this subset was 0.822. This model does not control for treatment duration, drug-class composition, or glaucoma severity.

Two-sided Wald p values and 95% CIs were obtained from the inverse analytical observed-information matrix. No Firth penalization was used in this supplementary analysis. The design was full rank, a linear-programming screen found no complete or quasi-separating direction, and the observed-information matrix was positive definite. The maximum absolute score was below 10^{-7}, and two numerical optimizers agreed within 10^{-5} per coefficient. Analysis was performed in Python with SciPy.

These estimates represent conditional associations among recorded preserved-exposure regimens. They do not establish an independent harmful effect of fixed-combination formulations. Non-DED denotes absence of a documented diagnosis, rather than a confirmed negative clinical assessment.
