# Supplementary material for: Documented Dry Eye Disease in Patients Receiving Topical Antiglaucoma Therapy: Associations with Treatment Characteristics
Source: Biomedicines. 2026 Oct 15;14(10):2344. doi: 10.3390/biomedicines14102344 (PMC13702091; doi:10.3390/biomedicines14102344)
Supplement: Supplementary file 1 [file biomedicines-14-02344-s001.zip › Supplementary_Table_S5.pdf]

Supplementary Table S5

Patient and documented DED counts by active-ingredient count and sensitivity analyses of predictor functional form.

A Patient and event counts

Active ingredients	Patients n	Documented DED n	No documented DED n	DED %
1	121	28	93	23.1
2	133	65	68	48.9
3	72	48	24	66.7
4	31	30	1	96.8

Total	Patients	Documented DED	No documented DED	DED %
All categories	357	171	186	47.9

DED denotes the clinical diagnosis documented at the index visit. No documented DED indicates absence of a recorded diagnosis rather than a confirmed negative clinical assessment. Percentages use the patient count within each category as denominator.

B Ingredient count in Model 1

Linear specification	Adjusted OR	95% CI	Wald p
Per additional ingredient	2.65	1.88–3.74	<0.001

Categorical specification	Adjusted OR	95% CI	Wald p
2 vs 1 ingredient	2.95	1.67–5.22	<0.001
3 vs 1 ingredient	4.55	2.07–9.99	<0.001
4 vs 1 ingredient	64.61	7.96–524.43	<0.001

Both specifications adjust for age, sex, and glaucoma subtype. The categorical specification uses one ingredient as reference. Likelihood-ratio $\chi^2 = 5.183$, df = 2; $p = 0.075$.

The categorical model did not clearly improve fit over the linear specification. This does not prove linearity. The four-ingredient estimate is imprecise because only one patient in that category lacked a documented DED diagnosis.

Supplementary Table S5 continued

C Ingredient count and treatment coefficients in the extended model

Linear count specification	Adjusted OR	95% CI	Wald p
Per additional ingredient	1.02	0.69–1.51	0.916
Exclusively PF regimen	0.0109	0.00141–0.0842	<0.001
Fixed-combination use	3.23	1.74–5.98	<0.001
Hypertension	1.76	1.01–3.05	0.046
Diabetes mellitus	1.65	0.85–3.18	0.139

Categorical count specification	Adjusted OR	95% CI	Wald p
2 vs 1 ingredient	0.0674	0.0187–0.24	<0.001
3 vs 1 ingredient	0.11	0.0283–0.46	0.002
4 vs 1 ingredient	1.58	0.14–17.38	0.710
Exclusively PF regimen	0.00134	0.000132–0.0135	<0.001
Fixed-combination use	4.17	2.21–7.88	<0.001
Hypertension	1.65	0.91–3.01	0.099
Diabetes mellitus	1.34	0.66–2.72	0.414

Both models additionally adjust for age, sex, and glaucoma subtype. Treatment duration is omitted, consistent with the original extended model. Ingredient categories use one ingredient as reference; other references are preserved-exposure regimen, no fixed-combination use, no hypertension, and no diabetes.

Likelihood-ratio $\chi^2 = 39.661$, df = 2; $p < 0.001$.

The categorical specification improved fit, showing that extended-model estimates were sensitive to ingredient-count functional form. The null coefficient per ingredient applies to the linear specification only. PF patients all received one ingredient and no fixed combination; the preserved-exposure one-ingredient stratum had 27/30 documented DED cases. Thus, the inverse categorical estimates must not be interpreted as protective effects of receiving two or three ingredients. Limited overlap, sparse outcomes, and treatment selection constrain these comparisons. These analyses use conventional maximum likelihood, not Firth penalization.

Supplementary Table S5 continued

D Treatment duration in Model 2

Linear specification	Adjusted OR	95% CI	Wald p
Per additional month	1.0099	1.0044–1.0154	<0.001

Likelihood ratio comparison	χ^2	df	p
Spline vs linear duration	0.726	1	0.394
Spline vs no duration	13.776	2	0.001

Spline contrast vs 65 months	Adjusted OR	95% CI
24 months	0.80	0.50–1.27
36 months	0.84	0.62–1.13
120 months	1.82	1.30–2.54

Both duration models adjust for age, sex, and glaucoma subtype. A restricted cubic spline with three knots at the 10th, 50th, and 90th percentiles (14.6, 65.0, and 154.4 months) includes one linear and one nonlinear basis term. Spline contrasts compare the stated durations with 65 months, the sample median, at the same adjustment-covariate values. Confidence intervals use the covariance of both spline coefficients.

There was no clear evidence of departure from linearity for treatment duration (p = 0.394). This supports retaining a linear duration summary but does not establish exact linearity. The overall spline association remained evident (likelihood-ratio p = 0.001).

Methods and interpretation

All models use the same 357 complete records. Estimates were obtained by conventional maximum-likelihood logistic regression. Two-sided Wald p values and 95% CIs use the inverse analytical observed-information matrix. Nested likelihood-ratio comparisons use two additional degrees of freedom for categorical versus linear ingredient count and one for spline versus linear duration. The overall duration test uses two degrees of freedom. No multiplicity adjustment was applied.

Numerical checks confirmed full-rank designs, positive-definite observed-information matrices, and no complete or quasi-separating direction in bounded linear-programming screens. Maximum absolute scores were below 10^{-7}; two optimization methods agreed within 10^{-5} per coefficient. Age and duration were rescaled for fitting without altering the reported contrasts.

These sensitivity analyses do not resolve limited exposure-group overlap, sparse PF events, unmeasured confounding, uncertain exposure timing, or selection of complete records. They assess predictor functional form and do not establish causal effects.

Software: Python 3.12.14, NumPy 2.3.5, pandas 2.2.3, SciPy 1.17.0. OR, odds ratio; CI, confidence interval; DED, dry eye disease; PF, preservative-free; df, degrees of freedom.
