# Supplementary material for: Documented Dry Eye Disease in Patients Receiving Topical Antiglaucoma Therapy: Associations with Treatment Characteristics
Source: Biomedicines. 2026 Oct 15;14(10):2344. doi: 10.3390/biomedicines14102344 (PMC13702091; doi:10.3390/biomedicines14102344)
Supplement: Supplementary file 1 [file biomedicines-14-02344-s001.zip › Supplementary_Table_S6.pdf]

Supplementary Table S6

Principal proportions of documented DED in the selected analytical sample.

Group	DED cases / n	DED %	95% Wilson CI %
Overall	171/357	47.9	42.8–53.1
Exclusively PF	1/91	1.1	0.2–6.0
Preserved exposure	170/266	63.9	58.0–69.4
One ingredient	28/121	23.1	16.5–31.4
At least two ingredients	143/236	60.6	54.2–66.6
2 ingredients	65/133	48.9	40.5–57.3
3 ingredients	48/72	66.7	55.2–76.5
4 ingredients	30/31	96.8	83.8–99.4

Percentages and 95% Wilson score intervals use the patient denominator within each stated group. Ingredient groups overlap with the aggregated ≥2 group and should not be summed across all rows. All proportions refer to the selected sample with complete ocular-surface records, not all glaucoma patients at the center. No documented DED does not imply a confirmed negative clinical assessment. PF, preservative-free; DED, dry eye disease; CI, confidence interval.
