# Supplementary material for: Documented Dry Eye Disease in Patients Receiving Topical Antiglaucoma Therapy: Associations with Treatment Characteristics
Source: Biomedicines. 2026 Oct 15;14(10):2344. doi: 10.3390/biomedicines14102344 (PMC13702091; doi:10.3390/biomedicines14102344)
Supplement: Supplementary file 1 [file biomedicines-14-02344-s001.zip › Supplementary_Table_S7.pdf]

Supplementary Table S7

All six correlations between treatment variables and ocular-surface measures.

Treatment variable	Ocular-surface measure	n	Spearman ρ	p
Active-ingredient count	TBUT	357	-0.904	<0.001
Active-ingredient count	Schirmer test	357	-0.858	<0.001
Active-ingredient count	OSDI	357	0.843	<0.001
Documented treatment duration	TBUT	357	-0.751	<0.001
Documented treatment duration	Schirmer test	357	-0.708	<0.001
Documented treatment duration	OSDI	357	0.691	<0.001

Two-sided Spearman rank correlations were recalculated for all six combinations using the same complete analytical dataset. Standard asymptotic p values were calculated using SciPy. No adjustment for multiplicity was applied. Pronounced clustering and tied values limit interpretation; the origin of repeated entries was not verified against source records. These correlations do not establish independent effects or causal relationships. TBUT, tear film breakup time; OSDI, Ocular Surface Disease Index.
