# Supplementary material for: Documented Dry Eye Disease in Patients Receiving Topical Antiglaucoma Therapy: Associations with Treatment Characteristics
Source: Biomedicines. 2026 Oct 15;14(10):2344. doi: 10.3390/biomedicines14102344 (PMC13702091; doi:10.3390/biomedicines14102344)
Supplement: Supplementary file 1 [file biomedicines-14-02344-s001.zip › Supplementary_Table_S8.pdf]

Supplementary Table S8

Sensitivity analysis excluding patients with previous oculoplastic surgery.

A Conventional maximum-likelihood model

Covariate	Adjusted OR	95% CI	Wald p
Age per year	1.00	0.97–1.03	0.868
Female vs male	0.85	0.49–1.45	0.545
Pseudoexfoliative vs POAG	0.55	0.27–1.12	0.100
Normal-tension vs POAG	0.40	0.13–1.26	0.116
Other subtype vs POAG	0.54	0.15–1.91	0.340
Active-ingredient count	1.00	0.66–1.51	0.994
Exclusively PF vs preserved-exposure regimen	0.011	0.001–0.087	<0.001
Fixed-combination therapy	3.57	1.90–6.71	<0.001
Hypertension	1.74	0.99–3.04	0.053
Diabetes mellitus	1.78	0.91–3.46	0.091

Both models include 345 patients and 159 documented DED events after excluding 12 patients, all with documented DED. The covariates match the original linear extended model: age, sex, glaucoma subtype, linear ingredient count, exclusively PF status, fixed-combination use, hypertension, and diabetes. Treatment duration is omitted. Reference categories are male, POAG, preserved-exposure regimen, no fixed combination, no hypertension, and no diabetes.

Two-sided Wald inference is used. For Firth estimation, standard errors use the inverse ordinary expected-information matrix evaluated at the penalized estimates; profile-penalized likelihood inference is not used. Full code and convergence diagnostics are supplied in Supplementary Code 1. Excluding this subgroup does not resolve sparse PF events, limited treatment overlap, or unmeasured confounding.

Supplementary Table S8 continued

B Firth-penalized model

Covariate	Adjusted OR	95% CI	Wald p
Age per year	1.00	0.97–1.03	0.872
Female vs male	0.85	0.50–1.46	0.560
Pseudoexfoliative vs POAG	0.56	0.27–1.14	0.110
Normal-tension vs POAG	0.42	0.14–1.30	0.134
Other subtype vs POAG	0.55	0.16–1.91	0.347
Active-ingredient count	1.00	0.66–1.50	0.996
Exclusively PF vs preserved-exposure regimen	0.018	0.003–0.095	<0.001
Fixed-combination therapy	3.38	1.81–6.29	<0.001
Hypertension	1.70	0.98–2.95	0.060
Diabetes mellitus	1.72	0.89–3.31	0.106

Both models include 345 patients and 159 documented DED events after excluding 12 patients, all with documented DED. The covariates match the original linear extended model: age, sex, glaucoma subtype, linear ingredient count, exclusively PF status, fixed-combination use, hypertension, and diabetes. Treatment duration is omitted. Reference categories are male, POAG, preserved-exposure regimen, no fixed combination, no hypertension, and no diabetes.

Two-sided Wald inference is used. For Firth estimation, standard errors use the inverse ordinary expected-information matrix evaluated at the penalized estimates; profile-penalized likelihood inference is not used. Full code and convergence diagnostics are supplied in Supplementary Code 1. Excluding this subgroup does not resolve sparse PF events, limited treatment overlap, or unmeasured confounding.
